# Influence of Pre-Treatment and Drying Methods on the Quality of Dried Carrot Properties as Snacks

**DOI:** 10.3390/molecules28176407

**Published:** 2023-09-02

**Authors:** Anna Ignaczak, Agnieszka Salamon, Jolanta Kowalska, Agata Marzec, Hanna Kowalska

**Affiliations:** 1 Department of Food Engineering and Process Management, Institute of Food Sciences, Warsaw University of Life Sciences, 159c Nowoursynowska St., 02-776 Warsaw, Poland; jolanta_kowalska@sggw.edu.pl (J.K.); agata_marzec@sggw.edu.pl (A.M.); 2 Institute of Agriculture and Food Biotechnology—State Research Institute, 36 Rakowiecka, St., 02-532 Warsaw, Poland; agnieszka.salamon@ibprs.pl

**Keywords:** blanching, osmotic enrichment, drying method, physicochemical properties, carrot snacks

## Abstract

The aim of the current research was to evaluate the effect of pre-treatment and drying methods on the properties of dried carrots. Carrots were blanched (B) (1 or 3 min) or osmotic dehydrated (OD) (15 or 30 min) and dried by either convection (CD), microwave-convection (MW-CD), microwave-vacuum (MVD), or freeze-drying (FD). FD carrots showed the highest dry matter content (93.6–95.8%) and the lowest water activity (0.24–0.38). MVD carrots had lower dry matter content (79.5–95.8%) and two times more water activity (0.447–0.637) than FD. The highest color difference (∆E) in relation to raw material was noted in MVD samples (22–35) and the smallest in CD and FD (7–18), mainly due to the increase in brightness of the dried carrot. In general dried MCD carrot samples were characterized by the highest max force (hardness) (21.6–42.5 N; on average 34.7 N) in the breaking test and the lowest hardness was observed in the CD (10.8 N) ones. Pre-treatment and drying caused a significant decrease in the content of carotenoids (2.0–2.7 times) and chlorophyll (2.7–4.5 times) compared to the fresh carrot but a retention or increase in the total content of phenolics and antioxidant activity, especially in microwave-vacuum-dried carrots with an increase of even 2.7–2.9 times compared to raw material. High phenolic content (195.6–277.4 mg GA/100 g d.m.) was found in pre-osmotic dehydrated samples, and lower phenolic content was found in blanched samples (110.7–189.6 mg GA/100 g d.m.). Significantly, the highest average antioxidant activity was found in microwave-vacuum-dried samples (228.9 µmol Trolox/100 g d.m.). The results of this study indicate that microwave-vacuum-drying as an alternative to freeze-drying, including in combination with thermal or osmotic treatment, is very promising for the production of dried carrot snacks.

## 1. Introduction

Modern consumers are increasingly aware of the impact of food on the proper functioning of the human body, which is why the market is seeing an increase in the popularity of low-processed food with the so-called “clean label”, i.e., only natural ingredients [1,2]. At the same time, due to an active lifestyle, most consumers eat their meals on the so-called “run”. In this case, vegetable snacks rich in dietary fiber, vitamins, and minerals are a good alternative. The presence of naturally occurring bioactive ingredients in these products means that this type of snack can be classified as functional food, having a scientifically proven beneficial effect on the functioning of the human body [3]. In 2016, the value of “healthy” snacks on the global market was $21.1 billion. Forecasts until 2025 indicate that the market for this product type will gradually grow by approx. 5.1% annually [4].

Carrots are a well-known root vegetable that has been cultivated around the world for over 2000 years. Carrot root comes in several varieties and is diverse in shape, size, and color. The most popular carrot is the orange root [5,6]. Carrots contain about 88.3% water, 1.0% protein, 0.2% fat, 4.7% sugars, 2.8% dietary fiber, and 1.0% minerals [7]. Orange carrots are also rich in vitamin A (5.01 mg/100 g), vitamin C (5.9 mg/100 g), thiamine (0.066 mg/100 g), riboflavin (0.058 mg/100 g), niacin (0.983 mg /100 g), vitamin B5 (0.273 mg/100 g), and vitamin B6 (0.138 mg/100 g) [7]. This vegetable is also rich in β-carotene (approx. 8.3 mg/100 g), which has a strong antioxidant and anticancer effect [7,8]. Due to their high water content, carrots are low in calories (41 kcal/100 g) [7]. Regular consumption of carrots has many health benefits, including the prevention of cardiovascular disease, diabetes, night blindness, cataracts, and cancer [8].

Bioactive ingredients in fruits and vegetables make them worth eating fresh. The need to use technologies to extend their durability also offers new valuable products. Drying is one of the oldest methods of food preservation. The quality of the dried material is determined by the appropriately selected drying method and parameters, i.e., time, temperature, flow rate of the drying agent, and the properties of the raw material, e.g., composition, shape, and structure [9]. Dried fruits and vegetables are still commonly obtained using simple drying methods, i.e., solar or air drying. However, these are time-consuming techniques, requiring a large amount of energy and affecting undesirable changes in quality, e.g., discoloration. Nevertheless, drying methods are constantly evolving. Currently, technologies have been developed that allow, above all, a decrease in the drying time and improve energy efficiency and quality characteristics of the obtained dried fruits and vegetables [10].

Convection drying is commonly used for fruit and vegetables because it does not require large financial outlays and complicated equipment. This process is characterized by low energy efficiency, at about 35% [9]. In addition, the material is exposed to a temperature for a long time (40–70 °C) and obtaining a moisture content of 20% involves a process that lasts several hours. The constant contact of the dried material with oxygen from the air also has a negative impact on the quality of the dried material. These factors are the reason for the deterioration of the sensory and nutritional quality of the product in comparison with the raw material. Qualitative features such as color, taste, aroma, shrinkage, consistency, structure, content of bioactive ingredients (vitamins and dyes), and antioxidant activity change [9,11,12]. In microwave drying, the material is heated throughout its volume. This facilitates the removal of water from the material, intensifying the drying process and improving the final quality of the product by limiting the degradation of bioactive compounds sensitive to higher temperatures [13,14]. Relatively new and effective microwave-vacuum drying technology allows for intensive evaporation of water from the surface of the dried material and obtaining the so-called puffing effect [11]. Drying using this technique results in a dried product qualitatively similar to freeze-dried material, e.g., in terms of the content of bioactive ingredients. Cui et al. [15] showed that the use of microwave-vacuum drying allowed the maintenance of a comparable content of carotenoids in carrot slices and chlorophyll in chive leaves, as in the case of freeze-drying. The short drying time of microwave-vacuum drying reduces costs and energy compared to lyophilization [16]. The production of vegetable snacks of different taste, shape, and consistency using various methods of pre-treatment, such as blanching and osmotic dehydration and drying, can become an interesting alternative to snacks available on the market. Both blanching and osmotic dehydration are becoming more and more common as complementary processing steps in the integrated food processing chain due to the energy and food-quality benefits [17,18]. It will certainly be great for consumers who do not like cooked vegetables, due to the change in sensory and nutritional values compared to the raw material [19].

The aim of this work was to examine the effect of blanching and osmotic dehydration, as a pre-treatment before drying, and drying methods such as convection, microwave-convection, microwave-vacuum, and freeze-drying on the physicochemical properties of dried carrots to obtain a vegetable snack.

## 2. Results

### 2.1. Influence of Pre-Treatment and Drying Method on Physical Properties of Dried Carrots

#### 2.1.1. The Dry Matter Content, Mass Loss, and Water Activity of Dried Carrots

The dry matter content of fresh carrots was about 9.4%. According to Mohammadi et al. [7], raw carrots contained about 10.0% dry matter, and according to Nagraj et al. [8] they contain 11.3%. These discrepancies may depend on the degree of maturity of the raw material, the variety, and the growing conditions. Drying carrot samples resulted in an increase in dry matter content of up to 77.1–96.5% (Table 1). The large variation in the values resulted in the fact that no statistically significant relationship was found between the type and time of pre-treatment and the method of drying on the content of dry matter in dried carrots. Nevertheless, some trends were identified.

Compared to the drying methods used and regardless of the pre-treatment, freeze-drying resulted in obtaining higher dry matter content (93.6–95.8%) (Table 1).

Noticeably higher dry matter content in the case of this drying method was characterized by the dried material without pretreatment, and the dried material subjected to blanching for 3 min was lower. The average dry matter content in dried materials obtained by the microwave-convection method was 84.5% (in the 79.1–92.8% range). In the study by Mohammadi et al. [7], air-dried carrots contained about 85.0% dry matter and freeze-dried carrots 91.6%. Despite the lack of a significant effect of pretreatment, a clear tendency to increase the drying effect of blanched and osmotically dehydrated samples was observed; the dry matter content increased to 91.0–96.5%. Lentas et al. [20] obtained higher contents of dry matter (95.0–96.0%) in dried mushrooms than in microwave-convective-dried mushrooms (85.4–89.3%).

Convection-dried (77.1–96.5%) and microwave-vacuum-dried (79.5–95.8%) carrot samples were characterized by similar average values of dry substance content, at the level of 89.8% (Table 1). A noticeably lower level of dry substance content among these samples was found only in convective-dried material without pre-treatment (77.1%), which may be due to the uneven drying of carrots in these conditions. On the other hand, samples after pretreatments of blanching and osmotic dehydration showed a higher dry matter content of 13.9–19.4%. Compared to osmotic dehydration, blanching had a more favorable effect on increasing the dry substance content in microwave-convective samples.

As a result of carrot drying, mass loss occurred (Table 1). Despite the variation in its value, ranging from 77.7 to 91.5%, the statistical analysis showed no significant effect of the drying method on the value of this parameter. The type of pre-treatment and its time had a statistically significant effect on the weight loss of dried carrots. Despite this, the values of mass loss in dried carrots without pre-treatment did not differ significantly from those that were pre-blanched. The time of blanching, i.e., 1 and 3 min, also did not significantly affect the value of this parameter. Nevertheless, both the lowest and the highest values of this index were recorded in freeze-dried samples. The lowest values, 77.7%, concerned samples pre-osmotically dehydrated for 30 min, and the highest, 91.5%, were found after pre-blanching for 3 min. Irrespective of the drying method, the use of the initial osmotic dehydration of carrots resulted in lower mass loss values. This may have been caused by a partial reduction in the water content and at the same time by the penetration of osmotic substances during osmotic pretreatment. In addition, along with the extension of the osmotic dehydration time, the loss of mass in dried carrots was lower, because the longer dehydration time contributed to a greater decrease in water content already during this treatment, and therefore the mass exchange during drying was not so intense. Nonetheless, no correlation (R^2^ = 0.018) was found between the dry matter content and mass loss in dried carrot samples. This could be the result of changes in the structure caused by the pre-treatment and drying method, especially the thermal and osmotic effects [1]. Thermal denaturation of the components of the cell wall and cell membranes, as well as its shrinkage and deformation as a result of mass transfer, could have caused water to be retained in its tissue and prevented carrot drying. Therefore, it will be useful to conduct research on the state of water in dried carrots under the discussed conditions.

The water activity in the dried carrot varied and ranged from 0.238 to 0.637 (Table 1). Statistical analysis showed that only the carrot drying method had a significant effect on water activity in dried carrots. The microwave-convective-dried carrot was characterized by the most diverse values of water activity, 0.297–0.621. The highest water activity in the case of this drying method was observed for carrot samples without pretreatment. This result could be caused by uneven drying of the material, and generally the difficulty in selecting drying conditions, especially at a high moisture content of the material [11,21]. Statistical analysis showed that the average water activity of samples dried by the microwave-convection method did not differ significantly from dried samples obtained by the microwave-vacuum one. Higher values of water activity (0.616–0.637), exceeding the required safety level of dried products (0.6), were observed in microwave-vacuum-dried samples without and after blanching, as well as in microwave-convection-dried samples without treatment (0.621). This may be due to difficulties in selecting drying parameters that would obtain dried material with favorable visual characteristics, at the required degree of drying of carrot samples. As shown by Kowalska et al. [11] microwave-vacuum drying, called hybrid or “puffing”, turned out to be very useful for obtaining dried fruit in the form of snacks (chips). In addition, the significant usefulness of the initial osmotic enrichment of plant materials, especially in juice concentrates or polyol solutions, was indicated. In addition to lowering the initial moisture content of the samples, this procedure influenced the development of sensory characteristics. These studies showed that the initial osmotic dehydration facilitated the implementation of the technological process. The increased content in enriching components susceptible to amorphous changes enabled the desired drying of the material and the permanent preservation of its structure. 

The lowest level of water activity (0.238–0.381) was observed in freeze-dried samples. These dried samples can be classified as food with low water content, and not very susceptible to the development of microflora [22]. Similarly, the lowest water activity in the case of freeze-dried pumpkin was obtained in the studies of Ciurzyńska et al. [23], as well as Kowalska et al. [11], who dried strawberries using various techniques and found that freeze-dried products were characterized by the lowest water activity. 

Dried carrot samples subjected to initial osmotic dehydration were characterized by a noticeably lower level of water activity compared to untreated and blanched samples. Extending the osmotic dehydration time did not cause significant changes in water activity (Table 1). These samples showed a safe level of water activity, i.e., below 0.6, at which, according to the literature, most microorganisms do not develop, and at values below 0.4, the activity of the Maillard reaction, lipid oxidation, hydrolysis, and enzymatic reactions decreases [22,24]. This may be due to the very mechanism of the osmotic dehydration process, consisting of a two-way exchange of mass. Water is removed from the dehydrated carrot, and the osmoactive substance from the hypertonic solution penetrates into this place [2]. As a result, this leads to an increase in the dry substance content of the carrot tissue and a decrease in the level of water activity.

#### 2.1.2. The Color Parameters and the Absolute Color Difference of Dried Carrot

**Figure 1:**
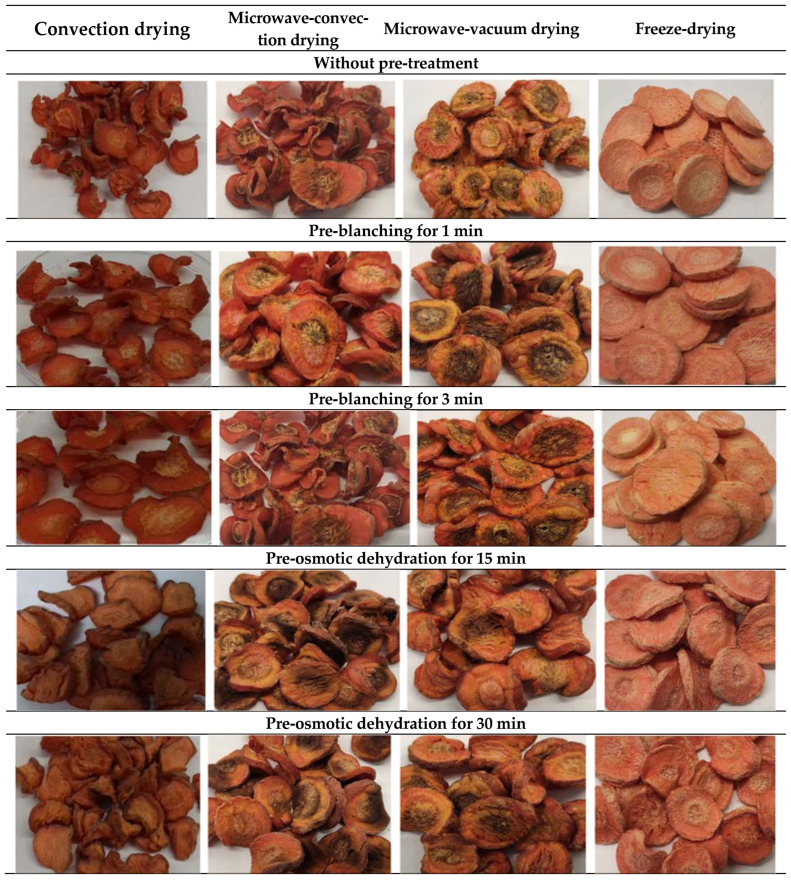
Pictures of dried carrots, depending on pre-treatment and drying method.

**Figure 2:**
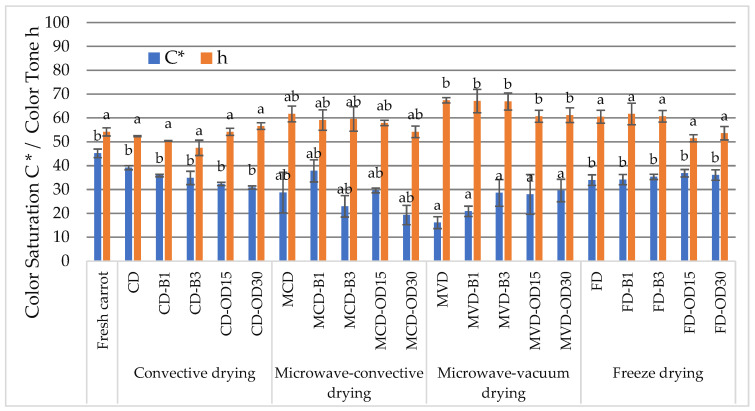
The effect of pre-treatment and drying method on values of C* and h color parameters of dried carrots. Different letters a, b (drying method)—homogeneous groups, show the statistical difference (*p* < 0.05). When all the data were in only one homogenous group A or A’, letters were omitted.

Color is one of the most important indicators of the quality of food products [24]. Very often it is the color that determines the choice and acceptance of a given product by the consumer [25]. At the same time, the color is susceptible to changes resulting from food processing. Heat treatment of vegetables (blanching) is often used to reduce color changes by inactivating polyphenol oxidase and peroxidase enzymes, as well as to improve other quality characteristics [17]. The CIE L*a*b* system is the most commonly used system for measuring the color of food products. The analysis of variance showed that only the method of drying had a significant effect on the color parameters of dried carrots. (Table 2, Figure 1 and Figure 2). One of the most important parameters determining the color of food products is the brightness L*. 

Convection drying without treatment increased the color brightness of carrot samples by about 6%, compared to the color of raw carrot (65.4), but to a lesser extent than freeze-drying (up to about 21%). The highest brightness (79.0) among all obtained dried fruits was characterized by freeze-dried carrots without pre-treatment. A slightly lower (73.8–78.0) value of the color parameter L* was found in freeze-dried products after initial blanching for 1 and 3 min (Table 2, Figure 1). On the other hand, the initial osmotic dehydration of carrots reduced the color brightness of dried carrots to the value of 59.6–62.3. It is worth emphasizing that the porous structure of dried material affects the angle of light reflection, which often results in an increase in the value of the L* color parameter, even though the human eye does not detect the brightening of the color [26].

A downward trend in the L* color parameter of the pre-blanched dried samples was observed, and the longer blanching time was conducive to a slightly greater darkening of the dried carrot samples (Table 2, Figure 1). Lentas and Witrowa-Rajchert [27] found that preliminary blanching of celery before drying caused a darkening of the color of the obtained dried product compared to the non-blanched dried product. Similarly, the pre-osmotically dehydrated and dried carrot was characterized by a significantly lower average value of the color parameter L* compared to samples without treatment or after initial blanching (Table 2).

A longer dehydration time favored the darkening of dried carrot samples, but no significant difference was found in the brightness of samples dehydrated for 15 and 30 min, whereas Kowalska et al. [11] showed that the initial osmotic dehydration of strawberries before freeze-drying caused a slight increase in color brightness. Zalewska et al. [27] found that convection drying of kiwi and bananas reduced the value of the L* color parameter compared to freeze-dried fruits. In the case of tangerines, the opposite relationship was shown, convective-dried carrots were lighter. 

The value of the color parameter L* of the freeze-dried samples differed significantly compared to the carrots obtained by microwave-convection and microwave-vacuum drying (Table 2). The average value of the L* color parameter of microwave-convective-dried MCD materials was lower (52.8) compared to convective-dried materials (62.4). The lowest average color brightness at the level of 48.6 was characteristic of microwave-vacuum-dried materials. Orikasa et al. [16] obtained a similar brightness in microwave-convection-dried tomatoes. Nonetheless, these dried MVD samples were significantly darker compared to freeze-dried carrots (70.5). According to the authors, the probable reason for obtaining such results was the longer time of convective drying and the high temperature of microwave drying.

Nowacka et al. [14] found that the use of microwaves with shorter impulses helps to improve the color of food products due to faster evaporation of water from the surface of the dried material. Color changes during food processing are related to their chemical composition [24]. The application of apple juice concentrate for the initial osmotic dehydration of carrots was aimed at reducing the moisture content, enriching the juice components, and improving the color of the dried carrot samples. Nevertheless, with a fairly intense orange color of carrots, no significant changes were observed compared to the control samples.

The color parameter a* characterizing the share of red color had different values in dried carrots, ranging from 6.3 to 26.3 (Table 2), but the statistical analysis showed a significant effect of only the drying method on the value of this parameter. The largest share of red color was found in convective-dried carrots (16.6–26.3). These samples differed the least from the color of fresh carrots (25.6). The share of red color in these samples was significantly higher (Table 2) than the ones obtained by the microwave-vacuum method, where the value of the color parameter a* ranged from 6.3 to 14.8. The red color of microwave-convective and freeze-dried carrots did not differ significantly. The average value of the parameter a* of the microwave-convection samples was 17.4, and in freeze-dried materials it was 19.3. The lack of a significant effect of the pre-treatment on redness resulted from the differentiation of its effect depending on the drying method. Freeze-dried samples after initial osmotic dehydration were characterized by a much higher redness index than in other dried materials, even close to the color of the raw material. Similar tendencies were observed in microwave-convective samples. On the other hand, blanching caused an increase in redness in convective and microwave-convective carrots. It can be assessed that the difficulties in the selection of microwave power and the duration of their operation had the greatest impact on the overall color quality of dried carrots assessed with redness. Despite the favorable texture, the samples dried unevenly and burned easily, especially in the core of the carrot slices. In the study by Zalewska et al. [27], convective-dried bananas and tangerines were characterized by higher values of the color parameter a* compared to freeze-dried fruits. Lentas and Witrowa Rajchert [26] found that blanching increased the share of the red color of dried celery roots.

As with the a* parameter, the values of the b* color parameter, the share of yellow, differed statistically only in terms of the drying method used (Table 2). The values of this indicator were very diverse and ranged from 15.2 to 34.1. Drying reduced the b* value of dried carrots by as much as 59% compared to the color of the raw material (36.8). A significantly higher share of yellow color was found in freeze-dried materials (29.4–30.1). On the other hand, a significantly lower value of this parameter was observed in the case of microwave-vacuum-dried carrots, which may result from the darker color of the carrot root core after drying. The values of the color parameter b* of dried carrots obtained by convection and microwave-convection methods did not differ significantly (Table 2). Wang et al. [28] used hot water treatment and the vacuum pulsed steam (VSPB) method to blanch carrots; they demonstrated a greater improvement in the color of the samples by increasing the redness and yellowness after using the VSPB steam method.

The obtained values of the absolute color difference, ∆E, in relation to the color of raw carrots (Table 2) and the photos of the obtained dried carrot (Figure 1) indicated a large variation of this indicator. Parameter ∆E values ranged from 5.2 to 34.8. The greatest difference in color in relation to the color of the raw material was found in microwave-vacuum-dried samples (22.0–34.8). The smallest differences in the color of dried carrots were noted for convection and sublimation samples. The average ∆E value of the color of convectively dried carrots was 11.4, and that of freeze-dried carrots was 12.5. This indicates differences noticeable to the untrained observer. Szadzińska et al. [29] found that green peppers dried by convection showed about 23% higher values of the absolute color difference than after microwave-convection drying. Differences in the value of ΔE may largely depend on the choice of the measurement point. Nahimana and Zhang [30], during the microwave-vacuum drying of carrot slices, showed differences in the color of the bark (outer part of the carrot root in cross-section) and the core (inner part of the carrot root in cross-section) in the case of both dried and fresh carrots. For example, the absolute color difference of the microwave-vacuum-dried materials within the bark was 91.2 ± 0.84 and that of the core was 68.7 ± 4.4. 

The color parameter C*, i.e., saturation, indicates the purity of the color. It can take a value from 0, meaning white and strongly subdued, to 100%, representing a pure, more intense color [31]. The values of the C* coefficient were very diverse and ranged from 16.1 to 39.2 (Figure 2). Drying contributed to the weakening of the color intensity of the dried carrot compared to the fresh product, with a value of 45.2. Statistical analysis proved that the drying method significantly affected this color parameter. The most significant differences occurred between the color of convective and freeze-dried carrots, and the microwave-vacuum-dried carrots. The values of this parameter in convective materials were 30.9–39.2, in freeze-dried materials were 34.0–36.9%, and in microwave-vacuum materials were 16.1–29.6%.

In the research of Ciurzyńska et al. [22], freeze-dried pumpkins were characterized by a higher level of color saturation compared to convective-dried ones.

The color tone h° shows how the color of the samples differs from white [31]. Dried carrots had different values of this index, ranging from 47.5 to 67.4°. These ranges correspond to colors from red to yellow. In most samples, the drying process caused an increase in the h° color parameter in relation to the color of fresh carrots, with a tone of 54.2° (Figure 2). The method of drying had a significant effect on the color shade of dried carrots. The h° values of carrots dried by convection (47.5–56.6°) and freeze-drying (51.5 to 61.7°) were at the lowest level. The highest value of the color coefficient h* was found in microwave-vacuum-dried carrot (60.5–67.4°). Łapczyńska-Kordon and Krzysztofik [25] showed that, depending on the raw material, the drying method influences the color shade of the dried material. Celery root, which contains much less sugar than apple (approximately 2 and 10%, respectively), was less prone to discoloration because the sugars did not caramelize during drying. The higher h° value of microwave-vacuum-dried materials can be explained by a faster drying process in comparison to convective and freeze-drying, during which color changes may occur due to temperature and oxygen. 

#### 2.1.3. The Texture Properties

**Figure 3:**
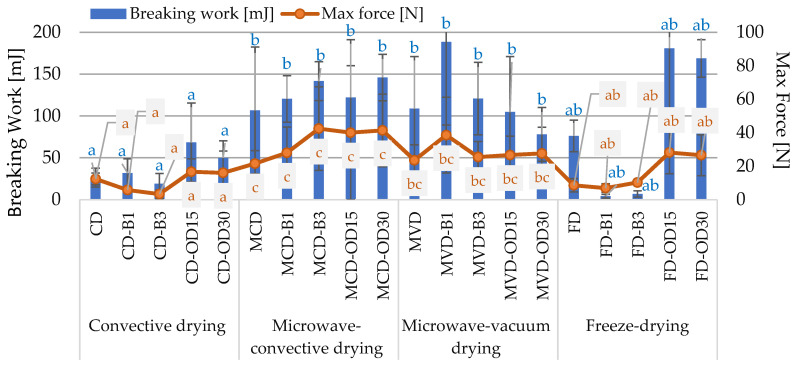
The effect of pre-treatment and drying method on max breaking force and breaking work of dried carrots. Different letters a, b, c—(drying method)—homogeneous groups, show the statistical difference at *p* < 0.05. When all the data were in only one homogenous group A, or A′, letters were omitted.

The texture is one of the most important and multidimensional aspects of the quality of food products, which largely depends on their chemical composition and structure. It is defined as a set of physical properties that can be felt by touch, related to deformation and fragmentation, i.e., changes caused by applied force. Dried carrots were characterized by quite diverse values of breaking energy (19.1–188.5 mJ; average 93.5 mJ) and lower maximum breaking force (3.3–42.5 N, average 22.5 N) (Figure 3). It should be emphasized that there were very extensive standard deviations of both indicators. Statistical analysis showed the effect of the drying method and the lack of effect of pre-treatment on the value of both the maximum force and the breaking work of dried carrot samples (Figure 3).

The highest strength, and thus the highest value of the maximum force (21.6–42.5 N; on average 34.7 N) necessary to destroy the samples in the breaking test, was characterized by microwave-convection-dried carrots (MCD). In the case of these dried samples, blanching and osmotic dehydration slightly increased the values of both indicators. Similarly, Lentas et al. [20] noticed that pre-blanched and microwave-convection-dried mushrooms required more force to be applied during the cutting test than convection and freeze-drying. This may be because carrots dried by microwave-convection were characterized by a significantly lower dry matter content than in dried carrots obtained by other drying methods (Table 1). Thus, the dried material contained the most water, which, acting as a plasticizer in food, contributed to the shape of a more rubbery structure, losing brittleness, and which required greater breaking force [14]. Microwave-vacuum-dried carrots were characterized by slightly lower breaking force, Fmax, in the range of 23.5–38.5 N, but higher breaking energy (78.0–188.5 mJ) than carrots dried by microwave-convection. However, there was no statistically significant difference between the data obtained for these types of dried carrots (Figure 3). Kowalska et al. [11] explained that during microwave-vacuum drying, the rapid transformation of liquid water into a gaseous state causes an increase in the volume of samples, the so-called “swelling” or “puffing” and reduces their shrinkage. As a result of drying, this state is fixed and reduces the collapse of the structure of the cell walls. The stable structure is also the result of the amorphous transformation of the components of the plant tissue. This was favored by higher temperature and the content of components susceptible to amorphous changes. Samples dried by convection and freeze-drying were characterized by a texture significantly more susceptible to breaking compared to microwave drying methods, but no statistically significant difference was found between them (Figure 3). For convective-dried material, the maximum breaking force ranged from 3.3 to 16.7 N and for freeze-dried products from 6.9 to 28.1 N. These dried materials were characterized by a correspondingly lower breaking load compared to the other dried materials, which could be due to the higher content of the dry matter in these samples (Table 1), and as a result, the dried material was more porous and brittle. During the breaking of these dried carrots, their internal structure was cracked, so the use of much less force allowed for the final cutting of the dried carrot.

The breaking work of dried carrots pre-osmotically dehydrated was characterized by quite diverse values (49.9–180.7 mJ). With the omission of MVD drying, osmotic dehydration contributed to a significant increase in the value of max force and breaking work, compared to untreated dried material. Freeze-dried materials subjected to initial osmotic dehydration were characterized by clearly higher values of the discussed parameter. Stępień [32] proved that the initial osmotic treatment increases the strength of microwave-vacuum-dried carrot slices. Ciurzyńska and Lenart [33], performing a compression test of freeze-dried, pre-dehydrated strawberries, observed that osmotic dehydration significantly increased the force and work of compression. Nevertheless, later research by Ciurzyńska et al. [34] showed an opposite relationship.

### 2.2. Influence of Pre-Treatment and Drying Method on Chemical Properties of Carrots 

#### 2.2.1. Content of Carotenoid and Chlorophyll Compounds

**Figure 4:**
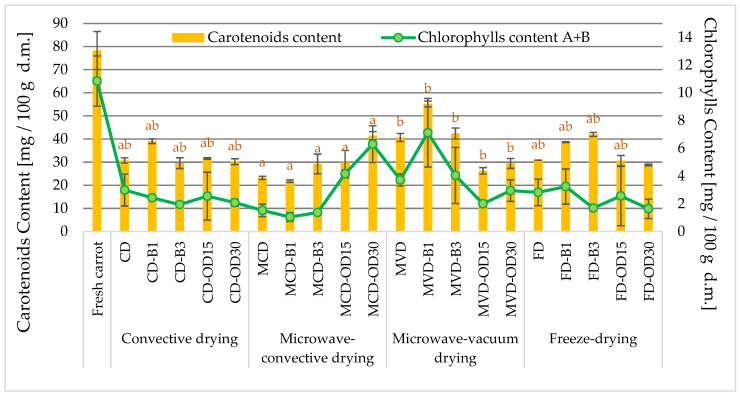
The effect of pre-treatment and drying method on the carotenoids and chlorophyll (A + B) content in dried carrots. Different letters a, b (type of drying method)—homogeneous groups, show the statistical difference (*p* < 0.05). When all the data were in only one homogenous group a or A, letters were omitted.

Carotenoids and chlorophylls are commonly found in natural plant dyes responsible for the characteristic red, orange, yellow, and green colors of raw materials of plant origin [35,36]. They have a strong antioxidant effect against free radicals and reactive oxygen species. In the group of compounds found in carrots, β-carotene has the highest provitamin A activity [37]. The total amount of carotenoids in fresh carrots was 78.3 ± 8.2 mg/100 g d.m., and chlorophyll was 10.8 ± 1.8 mg/100 g d.m. (Figure 4). A similar content of carotenoids (78.9 mg/100 g d.m.) in fresh carrots was found by Rawson et al. [38], and a slightly lower content (76.4 mg/100 g d.m.) was found by Cui et al. [15].

Both drying and pre-treatment significantly reduced the content of carotenoids and chlorophyll in dried carrots in comparison to fresh carrots. The content of carotenoids in the obtained dried carrot ranged from 21.8 mg/100 g d.m. for MCD-dried material up to 55.2 mg/100 g d.m. in MVD samples (Figure 4). On the other hand, the content of chlorophyll in dried carrots was in the range of 1.05–7.12 mg/100 g d.m. There was no significant effect of the method of drying and pretreatment on the content of chlorophyll in dried carrots (Figure 4). The obtained results were varied but some trends were observed. The average content of chlorophyll in the tested dried materials, from the lowest to the highest, amounting to 2.32, 2.40, 2.9, and 4.0 mg/100 g DM, was ranked according to the drying methods: freeze-drying FD ⇒ convection CD ⇒ microwave-convective MCD ⇒ microwave-vacuum MVD. 

A significant effect of the drying method on the content of carotenoids in dried carrots was found. The lowest average content of carotenoids was recorded in microwave-convective-dried carrots (29.1 mg/100 g d.m.). In convective- and freeze-dried materials, the content of carotenoids did not differ significantly. For convection-dried and freeze-dried carrots, it was on average 32.2 and 34.2 mg/100 g d.m., respectively. Microwave-vacuum-dried carrots were characterized by significantly higher values of this index, on average at the level of 38.8 mg/100 g d.m. This means that the microwave-vacuum method, especially after the initial application of blanching the carrots for 1 min, obtained the highest contents of carotenoids and chlorophylls (Figure 4).

Among the tested dried carrots, the lowest values of both indices were found in samples dried by the microwave-convective method after initial blanching, which were nearly 4 times lower than in the raw material. In the studies of Rawson et al. [38], blanching resulted in a decrease in the content of carotenoids by about 32% in both convection and freeze-drying methods. Wang et al. [28] showed that water and steam blanching increased the content of β-carotene in dried carrots by 0.5–0.9 mg/g d.m., compared to fresh carrots. They concluded that this may be due to the inactivation of enzymes and the destruction of the microstructure which leads to the extraction and alteration of the internal cell matrix during blanching.

Yusuf et al. [24] proved that dried colored carrot food products have a high content of nutrients and can be consumed by all age groups. These products were produced by a combination of osmotic dehydration of carrots of four colorful varieties (violet, orange, yellow, and white) in three juice concentrates (apple, chokeberry, and cherry) as well as convection and microwave-vacuum drying.

There was no significant effect of blanching time and osmotic dehydration as pretreatment on the content of carotenoids in dried carrots. Krzykowski et al. [39], analyzing the content of carotenoids in dried pepper subjected to initial blanching (1 and 3 min), showed a significant effect of blanching time on the content of carotenoids in dried pepper, with higher levels of carotenoids noted in the case of vegetables blanched for a shorter time. As demonstrated by Rawson et al. [39], this may be due to the destructive effect of blanching on the content of carotenoids during drying.

#### 2.2.2. Total Phenolic Content and DPPH Antioxidant Activity

**Figure 5:**
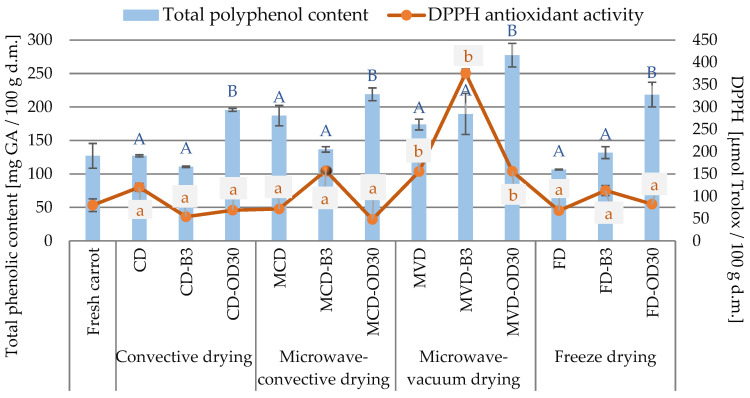
The effect of pre-treatment and drying method on total phenolic content and antioxidant activity (DPPH and ABTS) in dried carrots. Different letters a, b (type of drying method); A, B (type of pre-treatment)—homogeneous groups, show the statistical difference (*p* < 0.05). When all the data were in only one homogenous group a or A, letters were omitted.

The total phenolic content (TPC) in fresh carrots was 127.1 mg GA/100 g d.m. The content of these compounds was in the range of 106.5 mg GA/100 g d.m. for a lyophilized sample (FD) without pretreatment to 277.4 mg GA/100 g d.m. for microwave-vacuum-dried (MVD) samples subjected to prior osmotic dehydration (Figure 5). Statistical analysis showed no significant effect of the drying method on the content of these compounds. Only specific trends were observed. The average TPC content in the obtained dried materials, depending on the drying method, from the lowest to the highest value was as follows: convection method (CD—144.5 mg GA/100 g d.m.) ⇒ freeze-drying (FD—152.3 mg GA/100 g d.m.) ⇒ microwave-convection (MCD—180.9 mg GA/100 g d.m.) ⇒ microwave-vacuum (MVD—213.6 mg GA/100 g d.m.). The use of pre-treatment had a statistically significant effect on the average phenolic content of dried carrots. Pre-blanching of carrots for 3 min had a slight effect on the phenolic content, causing a slight reduction, while osmotic dehydration in all types of dried material resulted in significantly higher values compared to other samples, both those without pre-treatment and after blanching. Statistical analysis showed a significant effect of the drying method (*p* < 0.05) and no effect of the pre-treatment (*p* > 0.05) on the radical scavenging activity of DPPH (Figure 5). The microwave-vacuum-dried (MVD) materials had significantly higher average antioxidant activity (228.9 µmol Trolox/100 g d.m.) compared to fresh carrot and other drying methods. The average scavenging activity of DPPH radicals in the remaining dried carrots, depending on the drying method, did not differ significantly. From the lowest to the highest antioxidant capacity, the values were as follows in µmol Trolox/100 g d.m.: fresh carrot (80.1) ⇒ convective (CD—81.0) ⇒ freeze-drying (FD—87.6) microwave-convective (MCD—92.4) ⇒ microwave-vacuum (MVD—228.9).

With regard to the type of pre-treatment on the antioxidant activity of DPPH of dried carrots, certain tendencies were observed. The average radical scavenging activity of DPPH for pre-blanched samples was higher (175.0 µmol Trolox/100 g d.m.) than for dehydrated samples (88.8 µmol Trolox/100 g d.m.). Microwave-vacuum-dried (MVD) carrots subjected to initial osmotic treatment (30 min) were characterized by higher antioxidant activity (155.9 µmol Trolox/100 g d.m.) compared to all other types of dried material preceded by initial dehydration. A similar relationship was observed in the case of samples preceded by preliminary blanching. Microwave-vacuum-dried material (MVD) which was pre-blanched (3 min) had the highest antioxidant capacity (375.4 µmol Trolox/100 g d.m.) compared to other types of pre-blanched, dried material.

In the study by Gamboa-Santos et al. [40] dried carrots previously subjected to ultrasonic blanching preserved their total phenolic content and exhibited rehydration properties that were even better than those of the freeze-dried control. TPC values of convection-dried carrot samples (46 °C, 4.9 m/s, 7–9 h) after several traditional blanching procedures (steam, 2 min/98 °C or in boiling water; 1, 5, 40 min /60, 95 or 98 °C) and by ultrasound (60–70 °C, 10–15 min) were in the range of 1.312–1.524 mg GAE/g d.m. Raw carrot varieties are rich in phenolic acids and anthocyanins, and when dried in combination with osmotic enrichment carrots are also rich in flavan-3-ols and flavanols [24]. In the study by Wang et al. [28] blanching significantly increased the antioxidant capacity of convection-dried carrots compared to fresh samples. In addition, they showed that the antioxidant capacity was positively correlated with caffeic acid (r = 0.799) and ferulic acid (r = 0.791) under different blanching conditions, suggesting that phenols contribute significantly to the antioxidant capacity of carrots. A similar relationship was demonstrated in the research by Zhanga and Hamauzu [41] and Leja et al. [42] stating that it is the phenolic compound responsible for the antioxidant activity of carrot roots. The content of phenolic compounds and antioxidant properties of fresh carrots depends on their origin and root color, which translates into their content in dried carrots. Research conducted by Leja et al. [42] confirms that purple carrot roots contain, on average, about nine times more phenolic compounds than carrots with a different color of root.

## 3. Discussion

**Figure 6:**
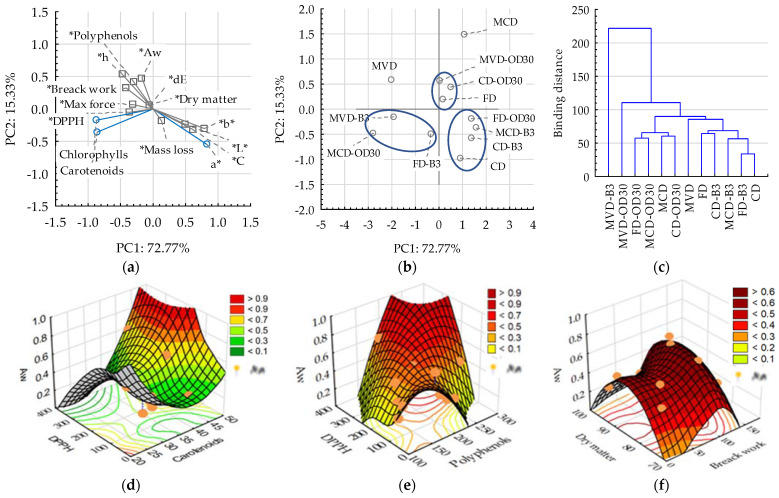
PCA and cluster analysis: (**a**) PCA loading plot of two principal components, (**b**) score plot presenting analyzed samples in terms of PC1 vs. PC2, (**c**) cluster analysis, (**d**–**f**) 3D surface plots. Markings: Aw—water activity; L*, a*, b*, C, h, and ΔE (dE)—color parameters. Blue lines indicate active data included in the PCA analysis, but —* and gray lines indicate additional data. The blue line on (**b**) concerns the separation of a group of data with a similar effect of pretreatment on the examined physicochemical indicators.

Principal component analysis (PCA) and cluster analysis were used to discuss the results. This made it possible to comprehensively determine the differences and similarities [43] of the analyzed dried carrot samples in terms of the applied pre-treatment and drying methods, as well as the tested indicators of physicochemical properties (Figure 6a).

The main components (PC1 and PC2) explained 88.1% of the variability of dried carrot properties. The content of dry matter, mass loss, absolute color difference, and breaking force as well as DPPH activity had little effect on PCA because they were arranged close to the middle of the graph. However, the mass loss was significantly negatively correlated (r2 = −0.79) with the total phenolic content (TPC), which could be related to their loss during the removal of water from dried carrots. As can be seen from the graph (Figure 6a), TPC was positively correlated with chlorophyll content, DPPH, and inversely (negatively) with dry matter content, which confirms that water migration from the samples during blanching, osmotic dehydration, or drying conditions may have caused the loss of these compounds. This can also be confirmed by the positively correlated water activity with DPPH (r2 = 0.58). Such results are interesting in comparison with the previously discussed ones, and their high variability (Figure 6d–f) did not result in clear dependencies and connections. The negative correlation of TPC with carrot color parameters was observed, excluding color tone h with a positive correlation. It was shown that the work of breaking was negatively correlated with mass loss but positively correlated with water activity. Appropriate tenderness of dried carrot snacks is desirable, and changes in tissue texture vary depending on the pre-treatment and selection of the drying method. Therefore, in the graph (Figure 6b) groups of samples with similar characteristics were separated. It is worth emphasizing the location of the carrot samples dried using the FD method, which is considered a model, and two other methods using osmotic dehydration for 30 min, MVD-OD30 and CD-OD30. As shown in previous studies [11], it is particularly advantageous to apply OD treatment before MVD to obtain a high-quality product, comparable or better than after drying with the FD method. According to the cluster analysis (Figure 6c), the most different sample was MVD carrots after the 3 min blanching. The obtained data could result from the drying conditions using individual methods. The greatest drying effect was found in the freeze-dried method, obtaining 93.6–95.8% dry matter content. Freeze-drying samples were carried out at 30 °C for about 24 h. Drying with the use of microwaves lasted much shorter, about 1 h, but the temperature could periodically reach 80 °C. Whereas, in the case of drying strawberries, Kowalska et al. [11] showed the high usefulness of the initial osmotic dehydration for microwave-vacuum drying, in the case of carrots this treatment facilitated the determination of drying parameters, but the dry substance content in dried carrots was comparable or lower than in control samples.

In conclusion, carrots are a relatively easy material to dry because all of the studied methods could obtain the required moisture level of dried carrots (dry matter content). When expecting high-quality dried carrots and not just a drying effect, carrot pre-treatment such as osmotic dehydration and blanching should be considered. This is particularly important in the case of the production of dried snacks from vegetables, the properties of which can be improved under the influence of enrichment and thermal treatment. The obtained results are interesting compared to those previously discussed, e.g., concerning the production of dried fruit snacks [11,22]. Due to the high variability of physicochemical indices and the lack of clear relationships and connections with input data (pre-treatment and drying method) (Figure 6e,f), there is a need to continue research, especially in connection with an in-depth analysis of the microstructure and sensory evaluation of dried carrot snacks.

## 4. Materials and Methods

### 4.1. Material and Experimental Procedure

The research material was carrots from the “Tadeusz Karaś” gardening farm, purchased in a large-area store.

Until the tests were carried out, the carrots were stored at a cooling temperature of about 4 °C and a relative air humidity of 85–90%. Vegetables of similar diameter (about 3 cm) were selected. The carrots were washed and then sliced into 5 mm slices with a Robot Coupe CL50 (Vincennes, France) fruit and vegetable slicer without removing the skin.

**Figure 7:**
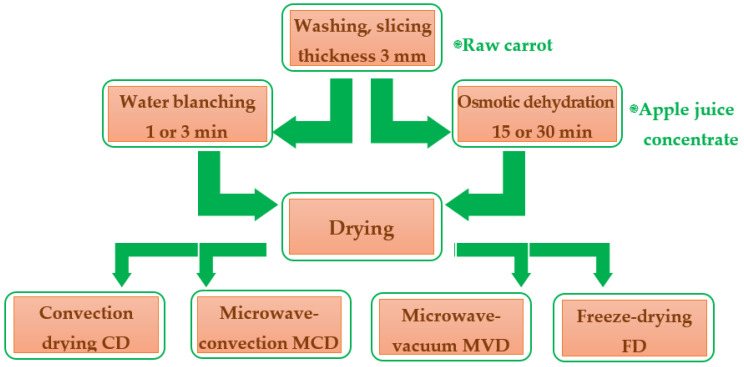
Scheme of the experimental procedure for drying carrots, including pre-treatment.

Carrot drying was carried out using such methods as convection, microwave-convection, microwave-vacuum, and freeze-drying, which were used after thermal treatment (blanching) or osmotic dehydration in apple juice concentrate (Figure 7). The tests were performed in triplicate.

### 4.2. Technological Methods

#### 4.2.1. Pre-Treatment Methods

Blanching was carried out by dipping about 100 g of carrots in boiling water. Two process time variants were used, i.e., 1 and 3 min. After blanching, the carrots were quickly cooled by immersing them in cold water for a while. Then the carrots were dried on filter paper and weighed on a technical balance (Type WPE 2000, RADWAG, Radom Poland) with an accuracy of 0.1 g.

The osmotic dehydration process was carried out in a water bath with a shaker (JW ELECTRONIC type T-OSM Warsaw, Poland) maintaining the set temperature. The osmotic solution was an apple juice concentrate with a concentration of 68 ± 0.2° Brix. The ratio of the mass of the sample to the mass of the osmotic solution used was 1:2. Osmotic dehydration was carried out at 70 °C for 15 and 30 min. After the dehydration process was completed, the samples were separated from the osmotic solution. The dehydrated carrots were rinsed in a stream of water for about 5 s and then dried on filter paper. The samples prepared in this way were weighed on a technical balance (Type WPE 2000, RADWAG, Radom, Poland) with an accuracy of 0.1 g.

#### 4.2.2. Drying Methods

Convection drying (CD) was carried out with a co-current airflow of 2 ± 0.1 m/s at a temperature of 60 ± 0.3 °C for 4 h in a laboratory convection dryer available at the Institute of Food Sciences at Warsaw University of Life Sciences (WULS).

The microwave-convection drying (MCD) process was carried out using a microwave-assisted convection dryer from PROMIS-TECH (Wrocław, Poland). Initially, an attempt was made to dry carrots in order to determine the appropriate parameters (time and microwave power) and to obtain high-quality dried material in terms of color and degree of drying. As a result, 100 g of carrot samples were dried for up to 80 min at 230 W microwave power. Drying was completed when the weight of the sample did not change or reached 10 g. The airflow was perpendicular to the layer of material with a velocity of about 3.5 m/s, and the outlet air temperature was 40 °C.

Carrot samples were dried in a microwave-vacuum dryer (MVD) by PROMIS-TECH (Wrocław, Poland). Initial drying tests were performed in order to select the parameters (pressure, microwave power, temperature and time) depending on the quality of the dried material obtained in terms of degree of drying and color. Four drying cycles, a temperature of 70 °C, and a reduced pressure of 5.0 kPa were used (Table 3). Fresh (control) and blanched carrot samples were dried using 550 W microwave power; while for drying samples dehydrated in the apple juice concentrate solution, the microwave power was 400 W. Using a K-type thermocouple (NiCr-Ni), the temperature of the steam removed from the dried material at the outlet from the drying chamber is measured. When the steam temperature rises above 70 °C, the microwaves turn off.

Freeze drying (FD) was preceded by freezing the raw material in a shock freezer with a cold air supply at −40 °C for 4 h. The frozen samples were dried in the Alpha 1–4 LSC freeze-dryer from Christ (Germany) for 24 h with the temperature of the heating shelves at 30 °C, the pressure inside the freeze-dryer chamber at 63 Pa, and the safety pressure at 103 Pa.

### 4.3. Physical Determination

#### 4.3.1. Dry Matter Content and Water Activity

The dry matter content of fresh and dried samples was determined by drying them in a laboratory dryer (WAMED SUP-65 WG, Warsaw, Poland) at 60 °C to a constant weight for about 24 h in accordance with AOAC 920.15, 2002. The vessels with/without samples were weighed on an analytical balance (ME54E/M, Metler, Warsaw, Poland) with an accuracy of 0.001 g. The measurement was performed in duplicate.

Water activity was determined with an AQUALAB CX-2 device (Decagon Devices Inc., Pullman, WA, USA). Measurements were carried out at the temperature of 23 ± 1 °C. The measurement was performed in duplicate, and the final result was the mean of the measurements [11].

#### 4.3.2. Color Parameters

The color of the dried carrots was measured with the Konica Minolta CR-300 colorimeter (standard observer CIE 2°, illuminate D65, measuring gap 8 mm) in the CIE Lab system. The measurement was performed in 5 replications. The mean of the measurements was taken as the result. The color of the dried samples was analyzed using the L* a* b* and L* C* h° systems and the absolute color difference ΔE, which was calculated with reference to the color of raw carrot as a standard.

#### 4.3.3. Texture Analysis

The study of the mechanical properties was carried out using a Texture Analyzer TA-HD plus (Stable Micro Systems, Godalming, UK) using a breaking test. The breaking force was recorded using the Texture Export computer program (for Windows). The dried samples were placed on wedge supports spaced 20 mm apart. The samples were tested with a load in its central part. The test was carried out at the head movement speed of 1 mm/s until the tested sample of dried carrot broke. The maximum force, Fmax [N], was determined as the point on the force-displacement curve of the head at which the destruction (breakage) of the tested material occurred of the tested material occurred. The breaking energy [mJ] was calculated using the area under the force-displacement curve of the head (mm) to achieve maximum force. The test was performed in at least ten replicates for each sample.

### 4.4. Chemical Determinations

Chemical determinations were carried out in cooperation with the accredited laboratory at the Institute of Agriculture and Food Biotechnology—State Research Institute in Warsaw, Poland. All determinations were performed at least in duplicate. Determinations of phenolic content and antioxidant activity were repeated at the Department of Food Engineering and Production Organization. The obtained results are the average of all determinations made in both institutes.

#### 4.4.1. Carotenoids and Chlorophyll A and B Content

Carotenoids and chlorophyll content in the dried samples were measured using the BECKMAN DU-530 spectrophotometer (Beckman, Wycombe, UK). The samples were ground in an analytical grinder (IKA A11 basic; IKA-Werke GmbH, Staufen, Germany), weighed, and added to 25 cm^3^ of 80% (*v*/*v*) to obtain an acetone solution extract. The samples were homogenized in an ULTRA-TURRAX T25 homogenizer (IKA-WERKE, Staufen, Germany). The homogenates were kept in the dark for 30 min at room temperature. Then they were centrifuged in a laboratory centrifuge MPW 375 (MPW-Med-Instruments, Warsaw, Poland) for 3 min at 7000 rpm. Two extracts were made for each sample. The measurements were made for chlorophyll A at wavelengths λ = 663 nm, for chlorophyll B λ = 647 nm, and at λ = 470 nm for carotenoids with the blank, which was 80% acetone solution. The determination was performed in duplicate. The content of chlorophyll and carotenoid pigments in the sample were calculated in mg per 100 g dry matter (d.m.) from the following equations [44]:

(1) $$C_{c}=\frac{1000\cdot A_{470}-1.82\cdot C_{A}-85.02\cdot C_{B}}{198}$$

(2) $$C_{A}=12.25\cdot A_{663}-2.79\cdot A_{647}$$

(3) $$C_{B}=21.50\cdot A_{647}-5.10\cdot A_{663}$$

(4) $$C_{A+B}=7.15\cdot A_{663}+18.71\cdot A_{467}$$

where:

C_c_—content of carotenoids in acetone extract [μg/mL];

C_A_—content of chlorophyll A in acetone extract [μg/mL];

C_B_—content of chlorophyll B in the acetone extract [μg/mL];

C_A+B_—content of chlorophyll (total A + B) in the acetone extract [μg/mL];

A_663_—absorbance of acetone extract measured at a wavelength of λ = 663 nm;

A_647_—absorbance of acetone extract measured at a wavelength of λ = 647 nm;

A_470_—absorbance of acetone extract measured at a wavelength of λ = 470 nm.

#### 4.4.2. Total Phenolic Content (TPC)

The total phenolic content was determined by spectrophotometric method with the use of the Folin–Ciocalteu reagent, which consisted of a colored reaction of phenolic compounds with this reagent [45]. To perform the test, 15% sodium carbonate (0.5 mL), distilled water (8.9 mL), acetone extract of the sample (0.5 mL; the extract was prepared in the same way as for the determination of carotenoids/chlorophylls), and 100 μL of Folin–Ciocalteu reagent were added to the tube. The sample was then mixed and incubated for 45 min in the dark (at room temperature). After this time, the absorbance was measured at the wavelength λ = 765 nm against the blank. When the measured absorbance of the sample was greater than 0.650 value, the sample was diluted with 80% (*v*/*v*) acetone solution. The determination was performed in duplicate. The total phenolic content was expressed as mg of gallic acid (GA) per 100 g dry matter (d.m.) of the sample.

#### 4.4.3. Antioxidant Activity (AA)

The antioxidant activity (AA) was determined using the spectrophotometric method with the DPPH radical [45]. For the preparation of samples, 2.4 mL of DPPH methanolic radical solution (60 μM) was used and 100 μL of acetone extract of the samples (the extract was obtained in the same way as for the determination of carotenoids/chlorophylls, Section 4.4.1) was added. The samples were mixed and incubated at room temperature for 30 min in the dark. After this time, the absorbance was measured at the wavelength λ = 515 nm against the blank. The acetone solution and DPPH solution were collected for the control sample. The blank was a sample containing methanol and 80% acetone.

The antioxidant activity (quenching/scavenging capacity) of the DPPH radical (% inhibition) was calculated:

(5) $$\%inhibition=\frac{A_{0}-A_{1}}{A_{0}}\cdot 100$$

where:

A_1_—absorbance of the DPPH radical with acetone extract from the sample;

A_0_—absorbance of the DPPH radical with acetone (control sample).

When the calculated inhibition was greater than the 95% value, the sample was diluted with 80% (*v*/*v*) acetone solution so that the absorbance value was linear over the range of the analyzed concentrations. The antioxidant activity (AA) based on the DPPH free radical scavenging ability of the extract was expressed as µM Trolox per 100 g of dry matter (d.m.) of the sample.

### 4.5. Statistical Analysis

The statistical analysis of the obtained results was performed with the use of Microsoft Excel and STATISTICA 13 PL programs. To determine the effect of pre-blanching and osmotic dehydration, as well as the drying method, on selected physicochemical indices, a one- or two-factor analysis of variance was carried out for the mean values from 3 repetitions. A Tukey’s HSD test was performed to determine homogeneous groups (post hoc test). Pearson’s correlation was also performed to investigate the relationship between the selected indicators. In addition, principal component (PCA) with classification and cluster analysis were performed.

## 5. Conclusions

Blanching and osmotic dehydration of carrots as well as drying by convection (CD), microwave-convection (MCD), microwave-vacuum (MVD), and freeze-drying (FD) had a varied effect on the quality of dried carrots in terms of dry substance content, mass loss, and water activity as well as texture, color, and chemical composition, i.e., the content of phenolic compounds, carotenoids, chlorophyll, and antioxidant capacity. The results showed that carrot blanching and osmotic dehydration can improve the quality of the dried product and preserve the color, phytonutrient content, and antioxidant capacity of carrots through short-term temperature action and osmotic enrichment in apple juice concentrate. Dried carrots were characterized by varied water activity in the range from 0.238 to 0.637; therefore, these dried foods can be classified as foods with low or medium water content, which ranged from the lowest for FD (0.238-0.381) to the highest (0.447–0.637) for MVD.

The MVD method is characterized by difficulty in selecting the drying parameters to obtain the appropriate water activity, but the use of thermal or osmotic treatment results in obtaining attractive, high-quality dried carrot snacks, especially in terms of chemical composition and antioxidant properties. Drying, including pre-treatment, contributed to a significant reduction in the content of carotenoids and chlorophylls. The average values of both indicators were 2.0–2.7 times and 2.7–4.5 times lower, respectively, compared to the raw material. On the other hand, dried carrots retained or increased the total phenolic content and antioxidant activity. The highest average values of these indices in microwave-vacuum-dried carrots even increased 2.7–2.9 times compared to raw carrots.

The use of pre-treatment, both thermal and osmotic, as well as microwave-vacuum drying, allowed the distinction in the quality of dried carrots in terms of the content of natural ingredients desired in the diet and antioxidant activity, as well as physicochemical properties comparable to other dried vegetables. Further in-depth studies are necessary to clarify the obtained results, especially in terms of changes in the microstructure, the state of water in dried food, as well as sensory evaluation. These studies will be useful in the selection of optimal conditions for the production of vegetable snacks, including on an industrial scale.

## Figures and Tables

**Table 1:** Dry matter content, mass loss, and water activity in dried carrot snacks.

Type of Dried	Dry Matter Content [%]	Mass Loss [%]	Water Activity [-]
Fresh carrot	9.41 ± 0.51	-	0.981 ± 0.006
CD	77.13 ± 1.90	85.83 ± 0.03 ^BB′C’^	0.362 ± 0.008 ^ab^
CD-B1	91.67 ± 1.73	90.10 ± 0.01 ^BC′^	0.464 ± 0.016 ^ab^
CD-B3	92.74 ± 2.70	88.63 ± 0.03 ^BC′^	0.447 ± 0.004 ^ab^
CD-OD15	96.49 ± 2.89	82.67 ± 0.03 ^AA′B′^	0.387 ± 0.006 ^ab^
CD-OD30	91.02 ± 0.44	78.72 ± 0.01 ^AA′^	0.363 ± 0.008 ^ab^
MCD	79.12 ± 1.71	85.83 ± 0.03 ^BB′C′^	0.621 ± 0.008 ^bc^
MCD-B1	92.82 ± 0.58	89.12 ± 0.03 ^BC′^	0.504 ± 0.004 ^bc^
MCD-B3	80.82 ± 0.08	90.20 ± 0.02 ^BC′^	0.508 ± 0.006 ^bc^
MCD-OD15	89.65 ± 4.07	82.01 ± 0.01 ^AA′B′^	0.503 ± 0.008 ^bc^
MCD-OD30	79.86 ± 1.90	80.64 ± 0.02 ^AA′^	0.297 ± 0.016 ^bc^
MVD	90.50 ± 2.74	87.14 ± 0.01 ^BB′C′^	0.616 ± 0.008 ^c^
MVD-B1	95.83 ± 0.22	90.00 ± 0.02 ^BC′^	0.637 ± 0.017 ^c^
MVD-B3	93.31 ± 0.52	84.15 ± 0.01 ^BC′^	0.626 ± 0.014 ^c^
MVD-OD15	79.49 ± 1.05	82.02 ± 0.01 ^AA′B′^	0.532 ± 0.014 ^c^
MVD-OD30	89.77 ± 0.98	81.28 ± 0.04 ^AA′^	0.447 ± 0.022 ^c^
FD	95.76 ± 0.48	88.98 ± 0.02 ^BB′C′^	0.294 ± 0.003 ^a^
FD-B1	95.00 ± 1.26	90.25 ± 0.01 ^BC′^	0.381 ± 0.004 ^a^
FD-B3	93.60 ± 0.56	91.47 ± 0.08 ^BC′^	0.345 ± 0.009 ^a^
FD-OD15	94.85 ± 0.83	82.06 ± 0.02 ^AA′B′^	0.238 ± 0.009 ^a^
FD-OD30	94.87 ± 0.42	77.70 ± 0.04 ^AA′^	0.253 ± 0.042 ^a^
**Two-way analysis of variance (ANOVA)**			
**Factors**	***p*** **-value**		
Type of pre-treatment (A,B)	0.3061	0.0000 *	0.1840
Drying method (a,b,c)	0.0850	0.9709	0.0006 *
**One-way analysis of variance (ANOVA)**			
Pre-treatment time (A′,B′,C′) of:			
Blanching	0.5667	0.0001 *	0.4137
Osmotic dehydration	0.8123	0.0216 *	0.3798

*—significant difference at a confidence level of 0.05; a, b, c, A, B, and A′, B′, C′—homogeneous groups; when all the data were in only one homogenous group a or A, letters have been omitted.

**Table 2:** Comparison of color parameters and the absolute color difference of dried carrot snacks, depending on pretreatment and drying method.

Type of Samples	Color Parameters			ΔE
	L*	a*	b*	
Fresh carrot	65.37 ± 0.8	25.58 ± 1.49	36.83 ± 1.14	-
CD	69.51 ± 3.42 ^bc^	26.27 ± 3.72 ^b^	31.34 ± 1.77 ^ab^	6.88 ± 0.05 ^a^
CD-B1	64.60 ± 2.10 ^bc^	23.45 ± 1.62 ^b^	26.87 ± 0.55 ^ab^	10.28 ± 0.09 ^a^
CD-B3	59.29 ± 3.53 ^bc^	25.37 ± 5.39 ^b^	26.75 ± 2.32 ^ab^	11.91 ± 0.19 ^a^
CD-OD15	59.81 ± 2.04 ^bc^	19.12 ± 1.64 ^b^	26.90 ± 0.84 ^ab^	12.98 ± 0.15 ^a^
CD-OD30	58.82 ± 1.46 ^bc^	16.55 ± 0.47 ^b^	26.73 ± 1.48 ^ab^	15.11 ± 0.09 ^a^
MCD	56.21 ± 6.60 ^ab^	13.50 ± 3.28 ^ab^	25.36 ± 6.76 ^ab^	19.10 ± 0.12 ^ab^
MCD-B1	60.98 ± 2.75 ^ab^	24.72 ± 5.44 ^ab^	34.11 ± 2.11 ^ab^	5.17 ± 0.09 ^ab^
MCD-B3	54.80 ± 3.00 ^ab^	24.80± 7.80 ^ab^	31.95 ± 4.01 ^ab^	11.51 ± 0.22 ^ab^
MCD-OD15	49.74 ± 3.72 ^ab^	13.33 ± 4.11 ^ab^	23.44 ± 5.31 ^ab^	23.85 ± 0.14 ^ab^
MCD-OD30	42.30 ± 2.93 ^ab^	10.59 ± 2.99 ^ab^	15.67 ± 4.24 ^ab^	34.79 ± 0.11 ^ab^
MVD	46.14 ± 2.46 ^a^	6.33 ± 0.83 ^a^	15.17 ± 2.13 ^a^	34.90 ± 0.17 ^b^
MVD-B1	46.51 ± 0.35 ^a^	9.10 ± 0.84 ^a^	18.58 ± 1.07 ^a^	31.11 ± 0.18 ^b^
MVD-B3	50.03 ± 5.00 ^a^	10.65 ± 1.69 ^a^	25.26 ± 5.77 ^a^	24.21 ± 0.17 ^b^
MVD-OD15	49.40 ± 5.67 ^a^	12.09 ± 3.06 ^a^	22.60 ± 7.23 ^a^	25.38 ± 0.14 ^b^
MVD-OD30	50.71 ± 3.97 ^a^	14.83 ± 3.30 ^a^	24.41 ± 5.49 ^a^	21.96 ± 0.08 ^b^
FD	79.01 ± 1.07 ^c^	17.11 ± 2.37 ^ab^	30.08 ± 1.33 ^b^	17.49 ± 0.10 ^a^
FD-B1	77.99 ± 1.54 ^c^	15.44 ± 3.34 ^ab^	29.55 ± 0.11 ^b^	17.85 ± 0.13 ^a^
FD-B3	73.75 ± 4.09 ^c^	17.09 ± 2.48 ^ab^	30.01 ± 0.56 ^b^	13.92 ± 0.25 ^a^
FD-OD15	62.33 ± 1.06 ^c^	23.64 ± 1.42 ^ab^	29.51 ± 1.33 ^b^	8.16 ± 0.07 ^a^
FD-OD30	59.58 ± 2.68 ^c^	23.19 ± 2.92 ^ab^	29.41 ± 1.06 ^b^	9.71 ± 0.08 ^a^
	**Two-way analysis of variance (ANOVA)**			
Factors	*p*-value			
Type of pre-treatment (A,B)	0.2983	0.6889	0.4964	0.7169
Drying method (a,b,c)	0.0002 *	0.0090 *	0.0408 *	0.0080 *
	**One-way analysis of variance (ANOVA)**			
Pre-treatment time (A′,B′) of:				
Blanching	0.7242	0.8058	0.7471	0.9198
Osmotic Dehydration	0.6557	0.8470	0.6610	0.7011

*—significant difference at a confidence level of 0.05; a, b, c, A, B, and A′, B′—homogeneous groups, when all the data were in only one homogenous group a or A, letters were omitted.

**Table 3:** Parameters of microwave-vacuum drying (MVD); code: fresh—control sample, B1 and B3—blanched for 1 and 3 min, OD15 and OD30—osmotically dehydrated for 15 and 30 min.

Parameters/ Cycles	Cycle I	Cycle II	Cycle III	Cycle IV Stabilization
**Pressure [kPa]**	5.0	5.0	5.0	-
**Microwave power [W]**	550—fresh, B1, B3 400—OD15, OD30	-	550—fresh, B1, B3 400—OD15, OD30	-
**Temperature [°C]**	70	-	70	-
**Time [s]**	270—fresh 300—B1, B3 210—OD15 200—OD30	270—fresh 300—B1, B3 240—OD15 210—OD30	270—fresh 300—B1, B3 210—OD15 200—OD30	270—fresh 300—B1, B3 240—OD15 210—OD30

## Data Availability

The data presented in this study are available upon request from the corresponding author.
